# Silencing of long noncoding RNA HOXA11-AS inhibits the Wnt signaling pathway via the upregulation of HOXA11 and thereby inhibits the proliferation, invasion, and self-renewal of hepatocellular carcinoma stem cells

**DOI:** 10.1038/s12276-019-0328-x

**Published:** 2019-11-22

**Authors:** Jun-Cheng Guo, Yi-Jun Yang, Jin-Fang Zheng, Jian-Quan Zhang, Min Guo, Xiang Yang, Xiang-Ling Jiang, Li Xiang, You Li, Huang Ping, Liu Zhuo

**Affiliations:** 1 Central South University Xiangya School of Medical Affiliated Haikou Hospital, Haikou, 570100 P. R. China; 2 Psychological Research Center of Hainan General Hospital, Haikou, 570311 P. R. China; 3 Department of Psychology, Hainan General Hospital, Haikou, 570311 P. R. China; 4 The Third People’s Hospital of Hubei Province, Wuhan, 430060 P. R. China; 5 Hengyang Maternal and Child Health Hospital, Hengyang, 421200 P. R. China

**Keywords:** Cancer genomics, TOR signalling

## Abstract

Hepatocellular carcinoma (HCC) is a major cause of cancer-related deaths, but its molecular mechanisms are not yet well characterized. Long noncoding RNAs (lncRNAs) play crucial roles in tumorigenesis, including that of HCC. However, the role of homeobox A11 antisense (HOXA11-AS) in determining HCC stem cell characteristics remains to be explained; hence, this study aimed to investigate the effects of HOXA11-AS on HCC stem cell characteristics. Initially, the expression patterns of HOXA11-AS and HOXA11 in HCC tissues, cells, and stem cells were determined. HCC stem cells, successfully sorted from Hep3B and Huh7 cells, were transfected with short hairpin or overexpression plasmids for HOXA11-AS or HOXA11 overexpression and depletion, with an aim to study the influences of these mediators on the self-renewal, proliferation, migration, and tumorigenicity of HCC stem cells in vivo. Additionally, the potential relationship and the regulatory mechanisms that link HOXA11-AS, HOXA11, and the Wnt signaling pathway were explored through treatment with Dickkopf-1 (a Wnt signaling pathway inhibitor). HCC stem cells showed high expression of HOXA11-AS and low expression of HOXA11. Both HOXA11-AS silencing and HOXA11 overexpression suppressed the self-renewal, proliferation, migration, and tumorigenicity of HCC stem cells in vivo, as evidenced by the decreased expression of cancer stem cell surface markers (CD133 and CD44) and stemness-related transcription factors (Nanog, Sox2, and Oct4). Moreover, silencing HOXA11-AS inactivated the Wnt signaling pathway by decreasing the methylation level of the HOXA11 promoter, thereby inhibiting HCC stem cell characteristics. Collectively, this study suggested that HOXA11-AS silencing exerts an antitumor effect, suppressing HCC development via Wnt signaling pathway inactivation by decreasing the methylation level of the HOXA11 promoter.

## Liver cancer: An RNA molecule that triggers tumor growth

A long RNA molecule promotes the growth of liver cancer cells through its inhibitory effects on gene regulation. The *HOXA11* gene controls cell proliferation and tissue development, and several studies have suggested that HOXA11-AS, an RNA that regulates this gene, may play a role in certain cancers. Researchers led by Min Guo at Hainan General Hospital in Haikou, China, have now obtained evidence linking HOXA11-AS to the growth of hepatocellular carcinoma cells. After determining that this RNA is consistently highly expressed in such cells, the authors demonstrated that it can stimulate cellular proliferation and invasive behavior through its suppressive effects on *HOXA11* and other genes. This inhibition results from HOXA11-AS-induced chemical modification of these DNA sequences. The authors hypothesize that this same mechanism could also contribute to growth of other tumor subtypes.

## Introduction

Hepatocellular carcinoma (HCC), which is acknowledged as the primary hepatic cancer, ranks as the six most common cancer worldwide and the fourth most frequently diagnosed cancer in China. In addition, HCC is the third most common cause of death due to cancer, both worldwide and in China^1,2^. Concerningly, in 2015, liver cancer emerged as not only the most frequently diagnosed cancer but also the most fatal cancer in individuals under 60 years of age^2^. HCC impairs normal liver function, leading to aberrant metabolism of glucose and lipids^3,4^. Multiple risk factors, including viral infections, liver cirrhosis, aflatoxin exposure, obesity, and diabetes, are related to HCC^5^. Hepatitis B and C viruses (HBV and HCV, respectively) account for ~80% of the viral infections linked with HCC^6^. Patients with liver cirrhosis arising from excessive alcohol consumption are at increased risk for HCC^7^. Hence, maintaining a healthy lifestyle may help prevent the occurrence of liver cancer. An improved understanding of molecular events involved in HCC pathogenesis can enable the understanding of mechanistic aspects and drive the potential development of novel therapeutic approaches.

The expression profiles of noncoding genes in HCC patients differ. In addition to microRNAs, long noncoding RNAs (lncRNAs) are important regulators of protein-coding genes^8,9^. In particular, dysregulation of lncRNAs is increasingly found in many diseases, especially cancers^10^. The *HOXA11* gene encodes transcription factors that control embryonic development events such as limb outgrowth^11^. Further work noted that *HOX* genes are involved in the control of lineage development in epithelial ovarian cancers^12^. HOXA11-AS (antisense) was demonstrated to act as a tumor suppressor in epithelial ovarian cancer by regulating HOXA11^13^. By contrast, in cervical cancer, HOXA11-AS overexpression was found to promote cell proliferation, migration, and tumor invasion and to play an oncogenic role^14^. HCC progression was shown to involve aberrant Wnt signaling, and Wnt signaling activation in HCC is documented^15^. *HOX* genes may affect the Wnt/β-catenin signaling pathway, as shown by HOXA13-mediated regulation of Wnt signaling in gastric cancer via its downstream effector CDH17^16^. In the present study, we explored a putative similar function of HOXA11-AS in HCC by investigating the role of HOXA11-AS modulation of HOXA1 expression and Wnt signaling in HCC stem cells.

## Materials and methods

### Ethics statement

The clinical data for all patients were obtained from medical records, and informed written consent was obtained from each participant. All experiments involving humans in the present study were conducted in strict accordance with the Declaration of Helsinki. The experimental animals were treated humanely, and all experimental procedures were approved by the Institutional Animal Care and Use Committee of the Central South University Xiangya School of Medicine Affiliated Haikou Hospital and the Psychological Research Center of Hainan General Hospital.

### Microarray-based in silico analysis

Information pertaining to HCC-related lncRNA expression profiles was downloaded from The Cancer Genome Atlas (TCGA) database (http://cancergenome.nih.gov/). The edgeR package coded in the R language was used to perform differential expression analysis of the transcriptomic data^17^. Differentially expressed lncRNAs in HCC were identified by comparing the RNA expression profiles from 374 samples of HCC tissues and 50 samples of adjacent normal tissues. False discovery rate (FDR) correction was employed for *p* values using the multitest package. Differentially expressed genes (DEGs) were identified using a screening threshold of FDR < 0.05 and |Log 2 fold change| > 1. The target genes of lncRNAs were predicted using the MEM website (https://biit.cs.ut.ee/mem/), and the STRING site (https://string-db.org/) was used for protein interaction analysis.

### Study subjects

Five HCC cell lines (SMMC-7721, HCCLM3, Hep3B, HepG2, and Huh7) and one immortalized human hepatocyte cell line (L02) were purchased from the Shanghai Institute of Cell Biology, Chinese Academy of Sciences (Shanghai, China). The expression of HOXA11-AS in the five HCC cell lines was determined by RNA isolation and quantitation.

Primary HCC tissues were collected from 116 HCC patients who underwent surgical resection at the Central South University Xiangya School of Medicine Affiliated Haikou Hospital and Psychological Research Center of Hainan General Hospital. These patients included 83 males and 33 females with a mean age of 49.28 years. The study subjects were followed up for 24 months, and survival analysis was performed using the Kaplan–Meier method. During the follow-up period, the death of patients was regarded as the endpoint; for surviving patients, the final follow-up time was considered the endpoint. The time interval from the date of surgery to the date of death was defined as the overall survival (OS) time. HCC tissues were collected in strict accordance with the general principles of specimen collection. A portion of the resected tissues was frozen at −80 °C, and the remaining part was fixed with 10% formalin and was further dehydrated in an automatic dehydrator and embedded in paraffin.

### Sorting of HCC stem cells

HCC cells were collected in the logarithmic growth phase and washed two times with phosphate-buffered saline (PBS), and the cell concentration was adjusted to 1 × 10^7^ cells/mL. These cells were then incubated with anti-CD133-PE (phycoerythrin) (566593, 1:500) and anti-CD44-FITC (fluorescein isothiocyanate) (555478, 1:500) antibodies for 30 min at room temperature. The antibodies were purchased from BD Biosciences (Franklin Lakes, NJ, USA). The fluorophore PE absorbs light at 490–560 nm and emits light at 595 nm, while the fluorophore FITC has an excitation wavelength of 495 nm and an emission wavelength of 519 nm. Subsequently, the cells were resuspended in 1 mL of PBS, filtered through a 40-μm sterile mesh, and placed on ice for stem cell sorting. The cells were incubated with isotype control antibody under the same conditions. A FACSAria II flow cytometer (BD Biosciences, Franklin Lakes, NJ, USA) was used to sort stem cells, and CD133^+^CD44^+^ cells were screened based on the fluorescence labeling characteristics of the different antibodies. Finally, the screened cells were collected into aseptic sorting tubes.

### Cell transfection and grouping

HCC cells collected in the logarithmic growth phase were seeded in 6-well plates at a density of 6–7 × 10^5^ cells/well, and cell transfection was conducted using a Lipofectamine 2000 Transfection Kit according to the manufacturer’s instructions. Twenty-five picomoles of plasmid and 10 μL of transfection reagent were mixed and added to each well at a final concentration of 10 pmol/mL. The transfected cells were then cultured at 37 °C in 5% CO_2_ for 48 h. Two HCC cell lines (Hep3B and Huh7) were used and assigned to the following groups: (1) sh-negative control (NC) (cells transfected with the short hairpin (sh)-NC plasmid); (2) sh-HOXA11-AS (cells transfected with the sh-HOXA11-AS plasmid); (3) oe-NC (cells transfected with the overexpression (oe)-NC plasmid); (4) oe-HOXA11-AS (cells transfected with the oe-HOXA11-AS plasmid); (5) oe-HOXA11 (cells transfected with the oe-HOXA11 plasmid); (6) sh-HOXA11-AS + sh-NC (cells cotransfected with the sh-HOXA11-AS and sh-NC plasmids); (7) oe-HOXA11-AS + oe-NC (cells cotransfected with the oe-HOXA11-AS and oe-NC plasmids); (8) oe-HOXA11-AS + oe-HOXA11 (cells cotransfected with the oe-HOXA11-AS and oe-NC plasmids); (9) sh-HOXA11-AS + sh-HOXA11 (cells cotransfected with the sh-HOXA11-AS and sh-HOXA11 plasmids); (10) dimethyl sulfoxide (DMSO) (cells treated with DMSO); (11) DNA methyltransferase (DNMT) inhibitor (5-aza-dC) (Sigma, St. Louis, MO, USA) (cells treated with the DNMT inhibitor); (12) methyltransferase SssI (M.SssI) (EM0821, Thermo Fisher, Process Instruments, Karlsruhe, Germany) (cells treated with M.SssI); (13) PBS (cells treated with PBS); (14) Dickkopf-1 (DDK1, a Wnt signaling pathway inhibitor, 100 μM) (cells treated with DDK1); (15) oe-HOXA11-AS + PBS (cells treated with PBS after transfection with oe-HOXA11-AS); and (16) oe-HOXA11-AS + DDK1 (cells treated with DDK1 after transfection with oe-HOXA11-AS). All target plasmids were purchased from Dharmacon (Lafayette, CO, USA).

### Tumorsphere formation assay

A total of 1 × 10^4^ HCC cells were seeded in low-attachment 96-well plates and cultured in serum-free Dulbecco’s modified Eagle’s medium (DMEM)-F12 medium containing 20 ng/mL epidermal growth factor and 20 ng/mL fibroblast growth factor-β. Semiquantitative liquid exchange was performed every 2 days. After culture for 10 consecutive days, the state of tumorsphere formation was photographed, and the number of tumorspheres was counted under a CKX4l inverted optical microscope (Olympus, Tokyo, Japan).

### RNA isolation and quantitation

Total RNA was extracted from cells and tissues using a TRIzol Kit (Invitrogen, CA, California, USA). The concentration and purity of the RNA were determined by a NanoDrop 2000 microvolume ultraviolet spectrophotometer (1011U, NanoDrop, Wilmington, DE, USA). RNA was reverse transcribed to complementary DNA using a TaqMan MicroRNA Assay Reverse Transcription Kit (4427975, Applied Biosystems, Foster City, CA, USA) according to the manufacturer’s instructions. Primers for HOXA11-AS, HOXA11, stemness-related transcription factors [Nanog, SRY-box-containing gene 2 (Sox2), octamer-binding transcription factor 4 (Oct4)], and cancer stem cell surface markers (CD133 and CD44) were designed and were then synthesized by Takara (Dalian, Liaoning, China) (Table 1). Reverse transcription-quantitative polymerase chain reaction (RT-qPCR) was performed using an ABI7500 Quantitative PCR Instrument (Applied Biosystems, Foster City, CA, USA), and relative quantification was performed using the 2^−ΔΔCt^ method^18^ with glyceraldehyde-3-phosphate dehydrogenase (*GAPDH*) as the internal reference gene. All experiments were repeated three times to obtain the mean values.

**Table 1:** Primer sequences used for RT-qPCR

Gene	Sequence (5′–3′)
*HOXA11-AS*	F: 5′-TGCCAAGTTGTACTTACTACGTC-3′
	R: 5′-GTTGGAGGAGTAGGAGTATGTA-3′
*HOXA11*	F: 5′-CGTGCGCGAAGTGACCTTCAGAGAGTAC-3′
	R: 5′-CCTGCCCACGGTGCTATAGAAATTGGAC-3′
*CD133*	F: 5′-TGGATGCAGAACTTGACAACGT-3′
	R: 5′-ATACCTGCTACGACAGTCGTGGT-3′
*CD44*	F: 5′-CCAAGATGATCAGCCATTCTGG-3′
	R: 5′-AAGACATCTACCCCAGCAAC-3′
*Oct4*	F: 5′-GACAACAATGAGAACCTTCAGGAGA-3′
	R: 5′-CTGGCGCCGGTTACAGAACCA-3′
*Sox2*	F: 5′-GGGAAATGGGAGGGGTGCAAAAGAGG-3′
	R: 5′-TTGCGTGAGTGTGGATGGGGATTGGTG-3′
*Nanog*	F: 5′-ATGAAGTGCAAGCGGTGGCAGAAA-3′
	R: 5′-CCTGGTG GAGTCACAGAGTAGTT C-3′
*GAPDH*	F: 5′-TCAGCAATGCCTCCTGCAC-3′
	R: 5′-TCTGGGTGGCAGTGATGGC-3′

*HOXA11-AS* homeobox A11 antisense, *HOXA11* homeobox A11, *HCC* hepatocellular carcinoma, *CSC* cancer stem cell, *Sox2* SRY-box 2, *Oct4* octamer-binding transcription factor 4, *GAPDH* glyceraldehyde-3-phosphate dehydrogenase, *RT-qPCR* reverse transcription-quantitative polymerase chain reaction, *F* forward, *R* reverse

### Western blot analysis

Total protein was extracted by using radioimmunoprecipitation assay cell lysis buffer (BB-3209, BestBio Inc., Shanghai, China). Extracted protein samples were separated by sodium dodecyl sulfate-polyacrylamide gel electrophoresis and transferred onto a polyvinylidene fluoride membrane under constant voltage conditions. The membrane was blocked with sealing solution for 1 h and was then incubated overnight at 4 °C with diluted rabbit polyclonal antibodies against HOXA11 (1:500, ab72591), CD133 (1:500, ab19898), CD44 (1:2000, ab157107), Nanog (1:1000, ab106465), SOX2 (1:1000, ab97959), and Oct4 (1:1000, ab137427) and with rabbit monoclonal antibodies against β-catenin (1:5000, ab32572), NKD1 (1:10000, ab133650), c-myc (1:1000, ab32072), and cyclinD1 (1:10,000, ab134175). On the following day, the membrane was further incubated with horseradish peroxidase-labeled secondary antibody, goat anti-rabbit immunoglobulin G (IgG) (1:2000, ab205718), at 37 °C for 1 h. All antibodies were purchased from Abcam (Cambridge, MA, USA). Finally, the membrane was washed three times with PBS (5 min/wash) and developed. The relative expression levels of the target proteins were calculated as the ratio of the gray value of the target protein band to that of the internal reference band (GAPDH). All experiments were repeated three times.

### Soft agar colony formation assay

A total of 1 mL of cell suspension and an equal volume of 0.7% agarose solution were diluted to a 0.35% agarose–cell mixture. Cells were plated at 1 × 10^4^ cells/100 cm^2^, with three parallel replicates for each group. After solidification of the agarose on the upper layer, 2–3 mL of culture solution was added on top of the surface (without crushing the agarose), followed by culture at 37 °C in 5% CO_2._ The culture medium was changed every 2–3 days, and cell culture was terminated at 1 month. The Petri dishes were observed and imaged under an inverted microscope to count the number of cell colonies that had formed. A cluster containing more than 50 cells was regarded as one cell colony.

### 5-Ethynyl-2′-deoxyuridine staining

Cell proliferation was measured using an 5-ethynyl-2′-deoxyuridine (EdU) Cell Proliferation Assay Kit according to the manufacturer’s instructions (ab146186, RiboBio Co., Ltd., Guangzhou, Guangdong, China). In brief, HCC cells collected in the logarithmic growth phase were stained with EdU solution for 2 h, fixed with PBS containing 4% paraformaldehyde for 15 min at room temperature, and incubated with 0.5% Triton X-100 at room temperature for 20 min. Next, the cells were incubated with Apollo®567 (RiboBio Co., Ltd., Guangzhou, Guangdong, China) (100 µL/well) for 30 min, and 100 μL of 0.5% Triton X-100 was added. Next, three visual fields were selected under a fluorescence microscope at high magnification, and EdU-stained cells (proliferative cells) and Hoechst 33342-stained cells (all cells) were counted separately. The proportion of EdU-stained (positive) cells was calculated using the following formula: EdU-positive staining rate = (the number of EdU-positive cell nuclei/the number of total cell nuclei) × 100%^19^.

### Transwell assay

Extracellular matrix gel (40 μL) was added onto the polycarbonate membrane in the apical portion of each 24-well Transwell chamber. The basolateral chamber was supplemented with 700 μL of precooled DMEM containing 10% fetal bovine serum. The Transwell chamber was then cultured in a 5% CO_2_ incubator under saturated humidity conditions at 37 °C for 24 h. After incubation, the cells on the Transwell chamber and basement membrane were wiped off with a wet cotton swab, and the remaining cells were fixed in methanol for 30 min, stained with 0.1% crystal violet for 20 min, and rinsed under running water. Finally, five visual fields were randomly selected under an inverted microscope, the number of invaded cells in each field was observed and counted, and a mean value was obtained.

### Fluorescence in situ hybridization

HOXA11-AS was localized in HCC stem cells using a fluorescence in situ hybridization (FISH) assay, which was performed according the manufacturer’s instructions (Ribo^TM^ lncRNA FISH Probe Mix (Red), RiboBio Co., Ltd., Guangzhou, Guangdong, China). First, a HOXA11-AS probe was designed based on the gene sequence of HOXA11-AS. HCC stem cells were incubated with 250 μL of hybridization solution containing 300 ng/mL probe at 42 °C overnight. On the following day, the cells were washed three times with phosphate-buffered saline containing Tween-20 (PBST) and sealed with an anti-fluorescence quenching agent. The cells were observed in five different visual fields under a fluorescence microscope (Olympus, Tokyo, Japan) and imaged.

### Methylation-specific polymerase chain reaction

The methylation status of the HOXA11 promoter region was detected by methylation-specific polymerase chain reaction (MS-PCR). In brief, genomic DNA was extracted from cells using a DNA Extraction Kit (Tiangen Biotech Co., Ltd., Beijing, China) following the standard kit instructions. DNA concentration and purity were assessed by ultraviolet spectrophotometry, and DNA was preserved at −80 °C. A total of 1 μg of DNA was modified by hydrosulfite and stored at −80 °C (for <1 month). The methylation-specific and nonspecific primers designed for the *HOXA11* gene are shown in Table 2. MS-PCR was conducted according to the protocol previously described by Herman et al.^20^. In brief, a total of 9 μL of MSP product was mixed with 1 μL of 10× loading buffer, electrophoresed on a 2.5% agarose gel containing 0.55 mg/L ethidium bromide, and imaged using a gel imaging system. If the CpG island in the HOXA11 promoter region was completely methylated, only the methylated primer could amplify the target band. If this CpG island was not methylated, only the unmethylated primer could amplify the target band. In the case of partial methylation, both pairs of primers could amplify the target band, and this condition was also regarded as methylation.

**Table 2:** Primer sequences used for MS-PCR

Length	Sequence (5′–3′)
Methylated primers	
115 bp	F: ATTTACGGTTTTAAATTTTGGTTTC
	R: AAATCTATTCTCCTAAAATCTCGCA
Unmethylated primers	
124 bp	F: TTATGGTTTTAAATTTTGGTTTTGA
	R: AAATCTATTCTCCTAAAATCTCACA

*MS-PCR* methylation-specific polymerase chain reaction, F forward, R reverse

### Chromatin immunoprecipitation

Cells were fixed with 1% formaldehyde at room temperature for 10 min to crosslink DNA and proteins. After crosslinking, the crosslinked DNA and proteins were randomly fragmented by ultrasonic treatment, and the supernatant was collected into three tubes. Next, anti-RNA polymerase II antibody (positive control), normal mouse IgG (NC) (ab109489, 1:100, Abcam, Cambridge, MA, USA), and an antibody specific for the target protein mouse anti-DNMT1 (ab183403, 1:50, Abcam, Cambridge, MA, USA) were added to the cells in the three tubes and incubated overnight at 4 °C. On the following day, the endogenous DNA–protein complexes were precipitated by using Protein A/G-Sepharose and briefly centrifuged to discard the supernatant. The nonspecific complexes were washed, decrosslinked at 65 °C overnight, and purified by phenol/chloroform to extract the DNA fragments. Finally, the binding of DNMT1 to the HOXA11 promoter region was detected by using primers specific for the HOXA11 gene promoter region^21^.

### RNA-binding protein immunoprecipitation

The binding of HOXA11-AS to DNMT1 was detected by using a RNA-binding protein immunoprecipitation (RIP) Kit (Millipore, Bedford, MA, USA)^22^. In brief, cells were lysed, and the lysate was collected. A portion of the lysate was used as input, and the other part was incubated with antibodies and magnetic beads for binding. The magnetic bead–antibody complexes were washed and resuspended in 900 μL of RIP Wash Buffer. The samples were placed on a magnetic stand to collect the magnetic bead–protein complexes for RNA extraction. The antibodies used in the RIP assay were as follows: anti-DNMT1 (ab183403; 1:50, Abcam, Cambridge, MA, USA), which was mixed at room temperature for 30 min, and IgG (ab109489, 1:100, Abcam, Cambridge, MA, USA), which served as the NC^23^.

### RNA pull-down

The 3′ end of purified RNA was biotinylated with a biotin RNA labeling mixture (Ambion, Austin, TX, USA). Next, 1 μg of labeled RNA was heated in RNA structure buffer [10 mmol/L Tris (pH 7), 0.1 mol/L KCl, and 10 mmol/L MgCl_2_] at 95 °C for 2 min, incubated on ice for 3 min, and incubated at room temperature for 30 min to allow the RNA to form a suitable secondary structure. Next, 3 μg of HCC stem cells were lysed with cell lysis buffer (Sigma, St. Louis, MO, USA) at 4 °C for 1 h and centrifuged at 12,000 × *g* for 10 min at 4 °C. The supernatant was collected and transferred to an RNase-free centrifuge tube. Subsequently, 400 ng of biotinylated RNA was mixed with 500 μL of RIP buffer and incubated with the cell lysate mixture for 1 h at room temperature. Streptavidin-coated magnetic beads were added to each binding reaction system and incubated at room temperature for 1 h. Finally, the cells were washed five times with RIP buffer and incubated with 5× loading buffer for 5 min at 95 °C. Western blot analysis was performed to measure the amount of eluted DNMT1 protein, with rabbit anti-DNMT1 (ab183403, 1:1000, Abcam, Cambridge, MA, USA) used as the primary antibody.

### In vivo limiting dilution assay

Cells were seeded in a low-attachment culture plate and cultured for 7 days. The resulting HCC stem cell spheres from each group were then collected by centrifugation in 10-mL glass centrifuge tubes. Next, the HCC stem cell spheres were washed once with normal saline and treated with 1 mL of 0.5% trypsin in an incubator at 37 °C for ~10 min (with flicking of the bottom of the plate every 2 min) to dissociate the cell spheres into single cells. Next, 3 mL of complete medium was added to stop further dissociation, and centrifugation was performed to collect the precipitated cells, which were resuspended in normal saline, titrated into a single-cell suspension, and counted. Next, different number of cells (5 × 10^3^, 1 × 10^4^, 5 × 10^4^, and 1 × 10^5^) were resuspended in 50 μL of normal saline, mixed with 50 μL of Matrigel matrix (1:1), and subcutaneously injected into BALB/c nude mice.

### Xenograft tumor in nude mice

In total, 246 BALB/c nude mice [male or female; age: 4–6 weeks; weight: 18–22 g; purchased from Hunan SJA Laboratory Animal Co., Ltd. (Changsha, Hunan, China)] were utilized in the experiment. The mice were raised in a specific pathogen-free environment and divided into three batches for the experiments. Mice in the first batch were subcutaneously injected with different number of cells (5 × 10^3^, 1 × 10^4^, 5 × 10^4^, and 1 × 10^5^) and subdivided into the Hep3B, Huh7, Hep3B-sphere, and Huh7-sphere groups, with six mice in each group. The tumor formation rate in these nude mice was determined 2 months later. Mice in the second batch were similarly subcutaneously injected with different number of cells (5 × 10^3^, 1 × 10^4^, 5 × 10^4^, and 1 × 10^5^) and subdivided into the oe-NC, oe-HOXA11-AS + oe-NC, oe-HOXA11-AS + oe-HOXA11, oe-HOXA11-AS + PBS, and oe-HOXA11-AS + DDK1 groups, each with six mice. The tumor formation rate was determined 2 months later. Mice in the third batch were assigned to the oe-NC, oe-HOXA11-AS + oe-NC, oe-HOXA11-AS + oe-HOXA11, oe- HOXA11-AS + PBS, and oe-HOXA11-AS + DDK1 groups, with six mice in each group. A total of 2 × 10^6^ cells were resuspended in 50 μL of normal saline, mixed with 50 μL Matrigel matrix (1:1), and subsequently inoculated subcutaneously into BALB/C nude mice. The tumor volume in each group was measured and recorded 2 weeks after inoculation.

### Statistical analysis

All experimental data were analyzed using SPSS 21.0 statistical software (IBM Corp., Armonk, NY, USA). Measurement data are presented as the means ± standard deviations. Differences between HCC and adjacent normal tissues were assessed using a paired *t* test. Comparisons of the data between two groups were performed using unpaired *t* tests, while those among multiple groups were performed using one-way analysis of variance or repeated-measures analysis of variance. A *p* value of <0.05 was considered to indicate statistical significance.

## Results

### HOXA11-AS is highly expressed in HCC tissues, cells, and stem cells

**Fig. 1:**
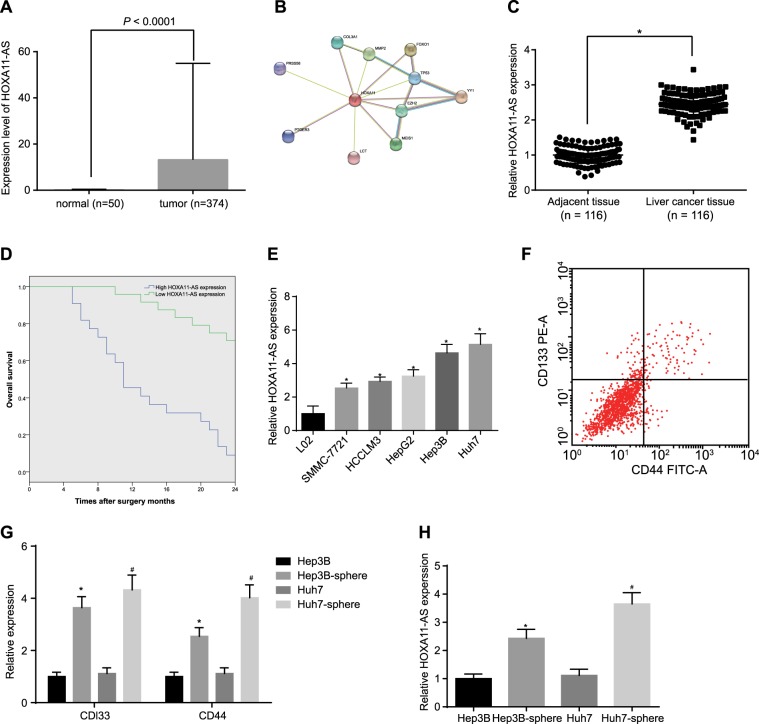
Highly HOXA11-AS expression is found in HCC tissues, cells, and stem cells. **a** The expression of HOXA11-AS as predicted by the TCGA database; *p < 0.05 vs. adjacent normal tissues; **b** the proteins interacting with HOXA11 as predicted by the STRING web-based platform; **c** the expression of HOXA11-AS in adjacent normal tissues and HCC tissues as measured by RT-qPCR; *p < 0.05 vs. adjacent normal tissues; **d** the correlation between HOXA11-AS expression and the prognosis of patients with HCC; **e** the expression of HOXA11-AS in the five HCC cell lines (MMC-7721, HCCLM3, HepG2, Hep3B, and Huh7) and the immortalized human hepatocyte cell line (L02) as measured by RT-qPCR; *p < 0.05 vs. L02 cells; **f** the sorting of CD133^+^CD44^+^ cells from Hep3B and Huh7 cells by flow cytometry; **g** the expression of CD133 and CD44 in Hep3B and Huh7 cells and in Hep3B and Huh7 spheres as measured by RT-qPCR; *p < 0.05 vs. Hep3B cells; ^#^*p* < 0.05 vs. Huh7 cells; **h** the expression of HOXA11-AS in Hep3B and Huh7 cells and in Hep3B and Huh7 spheres as measured by RT-qPCR; *p < 0.05 vs. Hep3B cells; ^#^*p* < 0.05 vs. Huh7 cells. All data are measurement data and are expressed as the means ± standard deviations. Comparisons of the data between two groups in **b** were performed with a paired *t* test (*n* = 116); comparisons among multiple groups were performed with one-way analysis of variance. All experiments were repeated three times

According to the TCGA database, HOXA11-AS was highly expressed in HCC (Fig. 1a). Coexpression of HOXA11-AS and HOXA11 was identified using the MEM platform. Protein interaction analysis using the STRING database predicted that HOXA11 interacts with FOXO1, which has been demonstrated previously^24^ (Fig. 1b). HOXA11 has been reported to inhibit the activation of the Wnt signaling pathway via the regulation of FOXO1^25,26^. RT-qPCR was performed to verify the expression pattern of HOXA11-AS in adjacent normal tissues and HCC tissues collected from 116 patients with HCC. The results showed that the expression of HOXA11-AS was higher in HCC tissues than in adjacent normal tissues and was also associated with the prognosis of patients with HCC (*p* < 0.05) (Fig. 1c, d). Next, the expression of HOXA11-AS in HCC cell lines (SMMC-7721, HCCLM3, HepG2, Hep3B, and Huh7) and an immortalized human hepatocyte cell line (L02) was measured. The results showed that the expression of HOXA11-AS was higher in the five HCC cell lines than in the L02 cell line. Among the HCC cell lines, the Hep3B and Huh7 cell lines showed the highest expression levels (*p* < 0.05) (Fig. 1e) and were thus selected for the following experiments.

To determine whether HOXA11-AS was also expressed at a high level in HCC stem cells, CD133^+/−^ and CD44^+/−^ cells were sorted from Hep3B and Huh7 cells by flow cytometry (Fig. 1f), and tumorsphere formation was employed to enrich HCC stem cells in Hep3B and Huh7 spheres. To confirm whether the obtained spheres were HCC stem cells, the expression of cancer stem cell surface markers (CD133 and CD44) in Hep3B and Huh7 spheres was measured by RT-qPCR (Fig. 1g). The results showed that CD133 and CD44 expression was significantly increased in Hep3B and Huh7 spheres compared with Hep3B and Huh7 cells (*p* < 0.05), preliminarily demonstrating the successful obtainment of HCC stem cells from Hep3B and Huh7 cells. Furthermore, the tumorigenicity of HCC stem cells in nude mice was assessed by subcutaneous injection of consecutive dilutions of Hep3B and Huh7 cells and Hep3B and Huh7 spheres. The results showed that the tumor formation rate in mice injected with Hep3B and Huh7 spheres was significantly higher than that in mice injected with Hep3B and Huh7 cells (*p* < 0.05) (Table 3), demonstrating that HCC stem cells were successfully isolated from Hep3B and Huh7 cells. To verify the expression of HOXA11-AS in HCC stem cells, its expression levels in Hep3B and Huh7 cells and in Hep3B and Huh7 spheres were measured. Hep3B and Huh7 spheres exhibited significantly higher HOXA11-AS levels than Hep3B and Huh7 cells (*p* < 0.05) (Fig. 1h). Together, these results suggested that upregulation of HOXA11-AS in HCC tissues, cells, and stem cells might be involved in the development and progression of HCC.

**Table 3:** Tumorigenicity of Hep3B and Huh7 spheres

Injected cells	Hep3B	Hep3B spheres	Huh7	Huh7 spheres
5 × 10^3^	1/6	2/6	0/6	2/6
1 × 10^4^	0/6	3/6	1/6	4/6
5 × 10^4^	2/6	4/6	2/6	5/6
1 × 10^5^	3/6	5/6	3/6	6/6
Total	6/24 (25.00%)	14/24 (58.33%)*	6/24 (25.00%)	17/24 (70.83%)^#^

Note: *p < 0.05 vs. Hep3B cells; ^#^*p* < 0.05 vs. Huh7 cells; the experiments were repeated six times

### Silencing of HOXA11-AS inhibits HCC stem cell self-renewal ability, invasion, and proliferation

As HOXA11-AS was found to be expressed at a high level in HCC stem cells, the effects of HOXA11-AS silencing on the biological characteristics of HCC stem cells were investigated via the introduction of sh-HOXA11-AS into Hep3B and Huh7 cells.

**Fig. 2:**
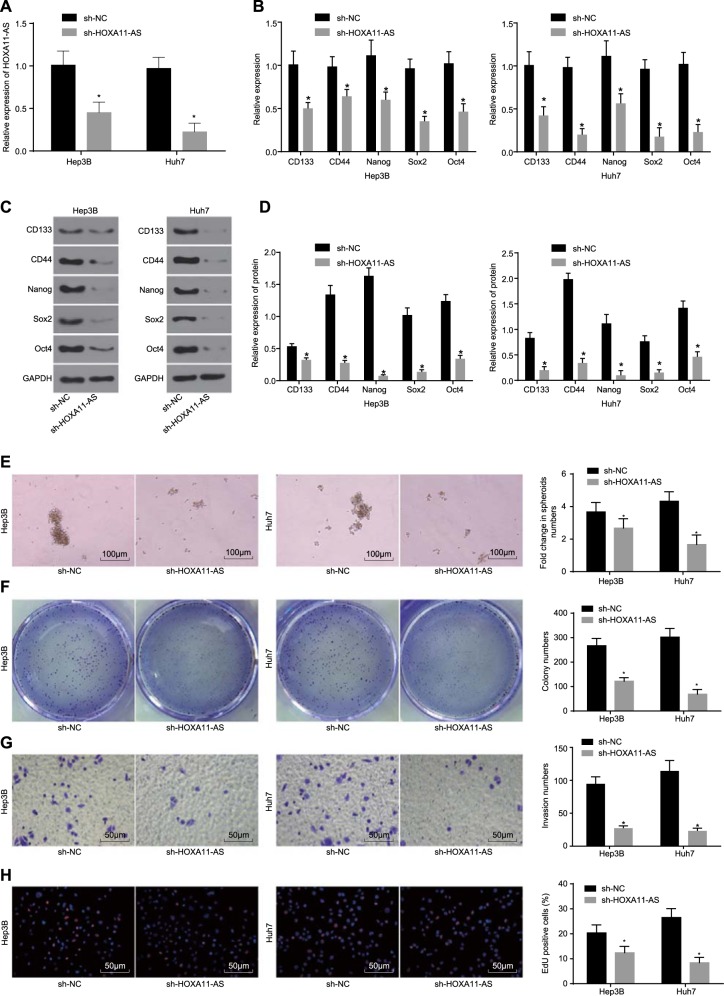
The self-renewal, invasion, and proliferation abilities of HCC stem cells are suppressed by HOXA11-AS silencing. **a** The interference efficiency of sh-HOXA11-AS in HCC stem cells as determined by RT-qPCR; **b** the expression of CD133, CD44, Nanog, Sox2, and Oct4 in HCC stem cells treated with sh-HOXA11-AS as measured by RT-qPCR; **c** gray value analysis of CD133, CD44, Nanog, Sox2, Oct4, and GAPDH bands; **d** the protein levels of CD133, CD44, Nanog, Sox2, and Oct4 in HCC stem cells following sh-HOXA11-AS transfection; **e** the tumorsphere formation ability of HCC stem cells treated with sh-HOXA11-AS as evaluated by a tumorsphere formation assay (scale bar = 100 μm); **f** the colony formation ability of HCC stem cells treated with sh-HOXA11-AS as evaluated by a soft agar colony formation assay; **g** the invasion ability of HCC stem cells treated with sh-HOXA11-AS as assessed by a Transwell assay (scale bar = 50 μm); **h** the proliferation ability of HCC stem cells treated with sh-HOXA11-AS as assessed by EdU staining (scale bar = 50 μm); *p < 0.05 vs. the sh-NC group. All data are measurement data and are expressed as the means ± standard errors. Comparisons of the data between two groups were performed with an unpaired *t* test. The experiments were repeated three times

The interference efficiency of sh-HOXA11-AS was evaluated, and it was shown that the expression of HOXA11-AS was significantly decreased in the sh-HOXA11-AS group compared with the sh-NC group (*p* < 0.05) (Fig. 2a). The messenger RNA (mRNA) and protein expression levels of CD133, CD44, Nanog, Sox2, and Oct4 in HCC stem cells were markedly reduced in the sh-HOXA11-AS group compared with the sh-NC group, as determined by RT-qPCR and western blot analyses (*p* < 0.05) (Fig. 2b, d). The effects of HOXA11-AS silencing on the self-renewal ability, invasion, and proliferation of HCC stem cells were assessed by tumorsphere formation assays, soft agar colony formation assays, Transwell assays, and EdU staining. As shown in Fig. 2e–h, the number of tumorspheres, cell colonies, invaded cells, and proliferative cells were decreased in the sh-HOCA11-AS group compared with the sh-NC group (all *p* < 0.05). These results provided evidence that the self-renewal, invasion, and proliferation capacities of HCC stem cells were reduced by HOXA11-AS silencing.

### HOXA11 is poorly expressed in HCC tissues and stem cells

**Fig. 3:**
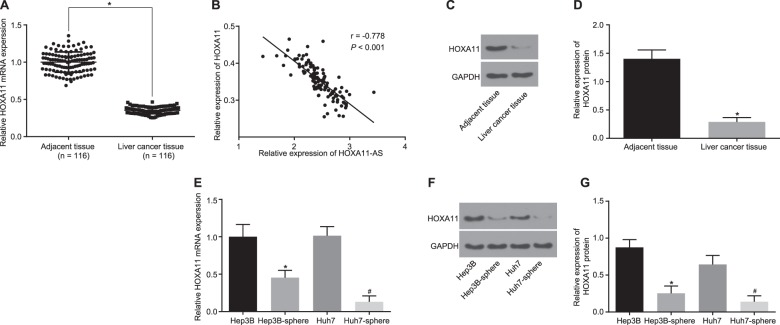
HOXA11 is downregulated in HCC tissues and stem cells. **a** The mRNA expression of HOXA11 in adjacent normal tissues and HCC tissues as measured by RT-qPCR; *p < 0.05 vs. adjacent normal tissues; **b** the correlation between HOXA11-AS and HOXA11 in tissues from HCC patients; **c** gray value analysis of HOXA11 and GAPDH protein bands; **d** the protein levels of HOXA11 in adjacent normal tissues and HCC tissues as measured by western blot analysis; *p < 0.05 vs. adjacent normal tissues; **e** the mRNA expression of HOXA11 in HCC stem cells as measured by RT-qPCR; *p < 0.05 vs. Hep3B cells; ^#^*p* < 0.05 vs. Huh7 cells; **f** gray value analysis of HOXA11 and GAPDH protein bands in Hep3B and Huh7 cells and in Hep3B and Huh7 spheres; **g** the protein levels of HOXA11 in Hep3B and Huh7 cells and in Hep3B and Huh7 spheres as evaluated by western blot analysis; *p < 0.05 vs. Hep3B cells; ^#^*p* < 0.05 vs. Huh7 cells. All data are measurement data and are expressed as the means ± standard deviations. Comparisons of the data between two groups in **a**, **b** were performed with a paired *t* test (*n* = 46); comparisons among multiple groups were performed with one-way analysis of variance. The experiments were repeated three times

To further clarify the regulatory relationship between HOXA11-AS and HOXA11, the expression levels of HOXA11 in HCC tissues, cells, and stem cells were measured by RT-qPCR and western blot analyses. HOXA11 was found to be downregulated in HCC tissues compared with adjacent normal tissues. Correlation analysis revealed that HOXA11-AS expression was negatively correlated with HOXA11 expression in HCC tissues (Fig. 3a–d). In addition, HOXA11 expression in Hep3B and Huh7 spheres was found to be lower than that in Hep3B and Huh7 cells. (Fig. 3e–g), suggesting that the expression of HOXA11 was decreased in HCC stem cells. Overall, these results suggested that the low expression of HOXA11 in HCC tissues might be involved in HCC progression.

### Overexpression of HOXA11 reduces HCC stem cell self-renewal ability, invasion, and proliferation

**Fig. 4:**
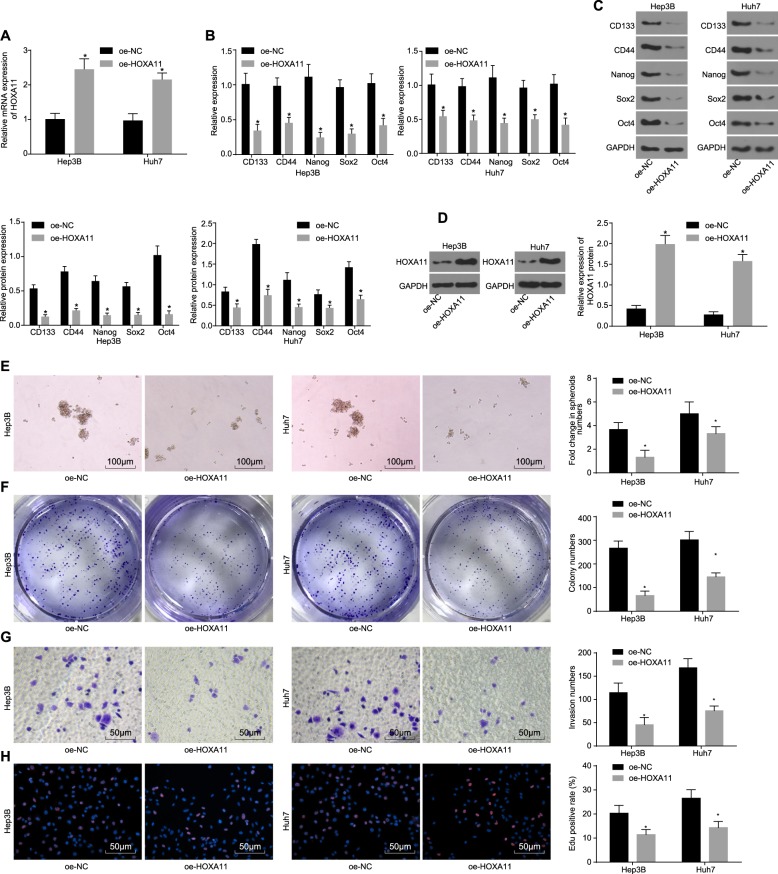
The self-renewal, invasion, and proliferation abilities of HCC stem cells are attenuated by HOXA11 overexpression. **a** The overexpression efficiency of HOXA11 in HCC stem cells as determined by RT-qPCR; **b** the expression of CD133, CD44, Nanog, Sox2 and Oct4 in HCC stem cells following oe-HOXA11 transfection as measured by RT-qPCR; **c** gray value analysis and expression of CD133, CD44, Nanog, Sox2, Oct4, and GAPDH protein; **d** the protein levels of HOXA11 in HCC stem cells following oe-HOXA11 transfection; **e** the tumorsphere formation ability of HCC stem cells following oe-HOXA11 transfection as evaluated by a tumorsphere formation assay (scale bar = 100 μm); **f** the colony formation ability of Hep3B and Huh7 cells following oe-HOXA11 transfection as evaluated by a soft agar colony formation assay; **g** the invasion ability of HCC stem cells following oe-HOXA11 transfection as assessed by a Transwell assay (scale bar = 50 μm); **h** the proliferation ability of HCC stem cells following oe-HOXA11 transfection as assessed by EdU staining (scale bar = 50 μm); *p < 0.05 vs. the oe-NC group. All data are measurement data and are expressed as the means ± standard errors. Comparisons of the data between two groups were performed with an unpaired *t* test, and comparisons among multiple groups were performed with one-way analysis of variance. All experiments were repeated three times

HOXA11 was found to be poorly expressed in HCC stem cells; therefore, the effects of HOXA11 overexpression on the biological characteristics of HCC stem cells were investigated by transfecting oe-HOXA11 into Hep3B and Huh7 cells. First, the transfection efficiency of oe-HOXA11 was validated by RT-qPCR and western blot analyses. The expression level of HOXA11 was significantly increased in the oe-HOXA11 group compared with the oe-NC group (*p* < 0.05) (Fig. 4a, d). Next, the expression levels of stem cell markers—CD133, CD44, Nanog, Sox2, and Oct4—in HCC stem cells were measured by RT-qPCR and western blot analyses and were found to be significantly lower in the oe-HOXA11 group than in the oe-NC group (*p* < 0.05) (Fig. 4b, c). Furthermore, the influence of HOXA11 overexpression on HCC stem cell self-renewal ability, invasion, and proliferation was assessed by tumorsphere formation assays, soft agar colony formation assays, Transwell assays, and EdU staining, as appropriate. The results revealed that HOXA11 overexpression reduced the number of tumorspheres (Fig. 4e), cell colonies (Fig. 4f), invaded cells (Fig. 4g) and proliferative cells (Fig. 4h) (all *p* < 0.05). These results suggested that the self-renewal, invasion, and proliferation capacities of HCC stem cells were attenuated by overexpression of HOXA11.

### HOXA11-AS suppresses the transcription of HOXA11 by recruiting DNMT1 to the HOXA11 promoter region

**Fig. 5:**
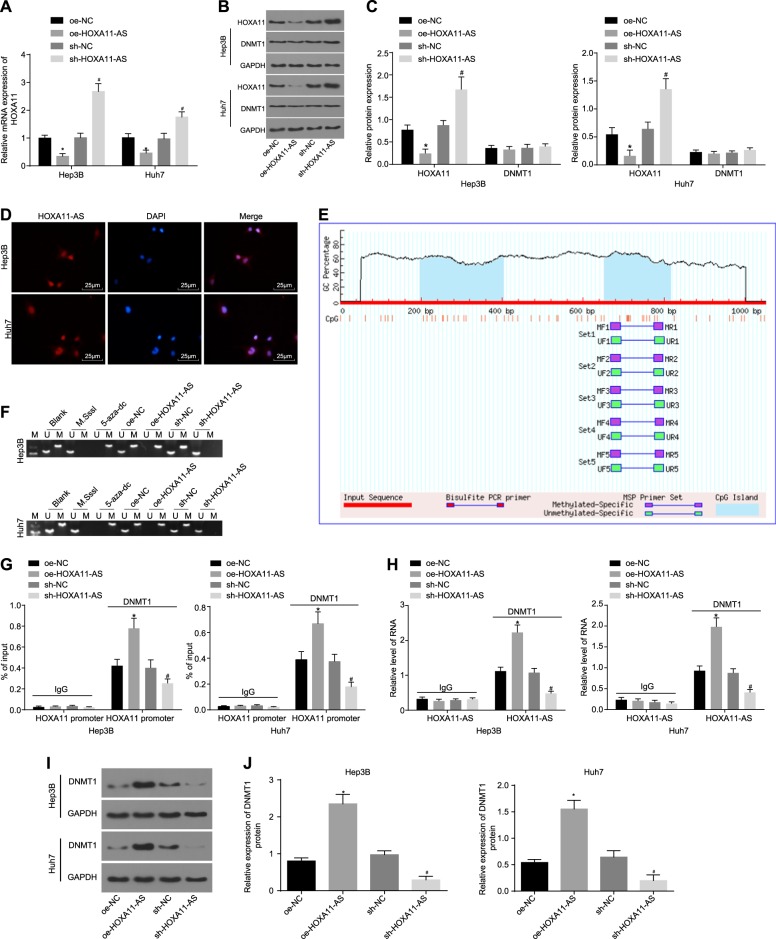
HOXA11-AS inhibits HOXA11 transcription by recruiting DNMT1 to the HOXA11 promoter region, resulting in its methylation. **a** The expression of HOXA11 in HCC stem cells as measured by RT-qPCR; **b**, gray value analysis of HOXA11, DNMT1, and GAPDH protein bands; **c** the protein levels of HOXA11 and DNMT1 in HCC stem cells as measured by western blot analysis; **d** the localization of HOXA11-AS in HCC stem cells as detected by FISH (scale bar = 25 μm); **e** the distribution of CpG islands in the HOXA11 promoter region; **f** the methylation level of the HOXA11 promoter as assessed by MS-PCR; **g** the status of DNMT1 enrichment in the HOXA11 promoter region in HCC stem cells as measured by ChIP; **h** the binding of DNMT1 to HOXA11-AS in HCC stem cells as evaluated by RIP; **i** RNA pull down to detect DNMT1 pulled down by HOXA11-AS; **j** Western bolt to detect DNMT1 protein expression, and the gray value of DNMT1 protein. *p < 0.05 vs. the oe-NC group; ^#^*p* < 0.05 vs. the sh-NC group. All data are measurement data and are expressed as the means ± standard errors; comparisons of the data between two groups were performed with an unpaired *t* test; comparisons among multiple groups were performed with one-way analysis of variance. The experiments were repeated three times

The relationship between HOXA11-AS and HOXA11 was studied by quantification of HOXA11 expression in HCC stem cells with overexpression or silencing of HOXA11-AS. It was revealed that the expression of HOXA11 was significantly downregulated in the oe-HOXA11-AS group compared with the oe-NC group, but was upregulated in the sh-HOXA11-AS group compared with the sh-NC group (both *p* < 0.05); however, HOXA11 expression showed no pronounced change in the DNMT1 expression group (*p* > 0.05) (Fig. 5a–c).

The regulatory mechanism between HOXA11-AS and HOXA11 was further studied. The results of the FISH assay showed that HOXA11-AS was expressed mainly in nuclei (Fig. 5d). HOXA11-AS has been reported to be capable of recruiting DNMT1 to gene promoter regions^27^; thus, it was hypothesized that HOXA11-AS might affect HOXA11 transcription via methylation of the HOXA11 promoter region. The distribution of CpG islands in the HOXA11 promoter region was predicted through an online website (http://www.urogene.org/cgi-bin/methprimer/methprimer.cgi), and it was found that a large number of CpG islands were distributed in the HOXA11 promoter region (Fig. 5e). Subsequently, the methylation levels of the HOXA11 promoter in HCC stem cells were measured by MS-PCR. The results showed that the methylation level of the HOXA11 promoter was notably higher in the M.SssI group, but distinctively lower in the 5-aza-dc group than in the blank group. A higher methylation level of the HOXA11 promoter was found in the oe-HOXA11-AS group than in the oe-NC group, while a lower methylation level of the HOXA11 promoter was found in the sh-HOXA11-AS group than in the sh-NC group (Fig. 5f) (*p* *<* 0.05). The results of MS-PCR suggested that HOXA11-AS can promote methylation of the HOXA11 promoter.

Next, the binding status of DNMT1 to the HOXA11 promoter region in HCC stem cells was determined by chromatin immunoprecipitation (ChIP). DNMT1 enrichment was higher in the oe-HOXA11-AS group than in the oe-NC group, but was lower in the sh-HOXA11-AS group than in the sh-NC group (Fig. 5g) (*p* < 0.05). In addition, the binding of DNMT1 to HOXA11-AS was assessed by RIP. The amount of DNMT1 binding to HOXA11-AS was significantly higher in the oe-HOXA11-AS group than in the oe-NC group, but was lower in the sh-HOXA11-AS group than in the sh-NC group (Fig. 5h) (*p* < 0.05). Furthermore, the recruitment of DNMT1 by HOXA11-AS was determined by RNA pull-down. A significantly higher amount of DNMT1 was recruited by HOXA11-AS in the oe-HOXA11-AS group than in the oe-NC group, while this amount was notably lower in the sh-HOXA11-AS group than in the sh-NC group (Fig. 5i, j). These results suggested that HOXA11-AS inhibits HOXA11 transcription by recruiting DNMT1 to the HOXA11 promoter region, thereby resulting in HOXA11 gene methylation.

### Silencing of HOXA11-AS inactivates the Wnt signaling pathway by upregulating HOXA11

**Fig. 6:**
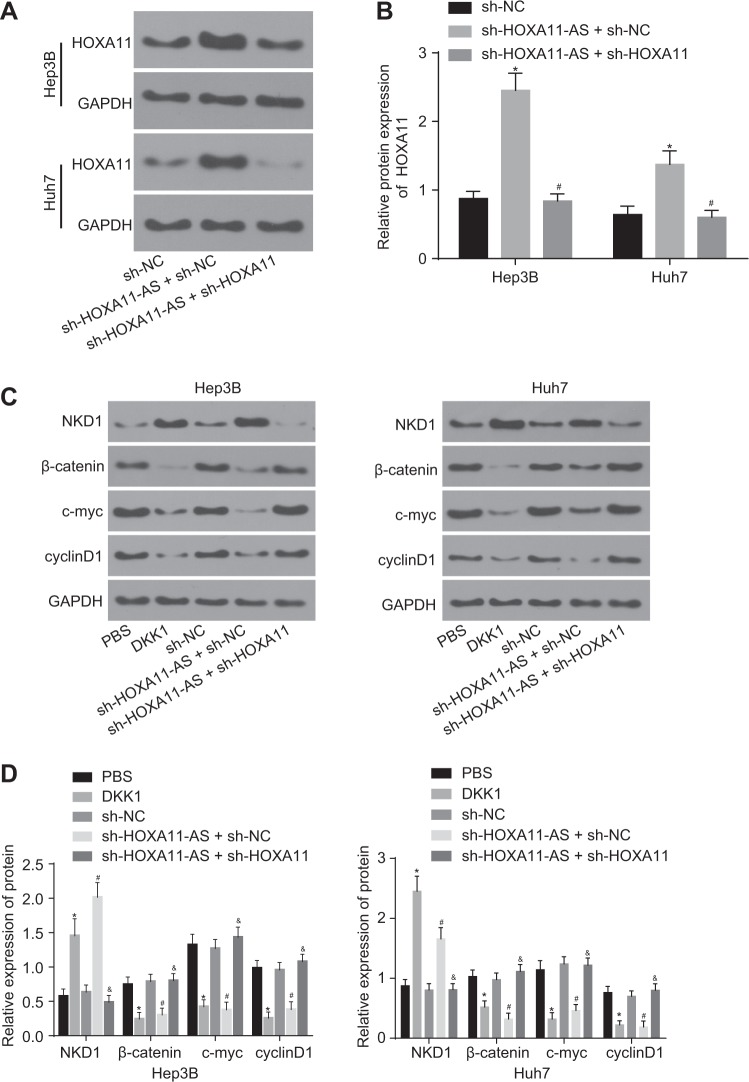
HOXA11-AS silencing suppresses Wnt signaling pathway activation by promoting HOXA11 expression in HCC stem cells. **a** Gray value analysis of HOXA11 and GAPDH protein bands; **b** the protein levels of HOXA11 as determined by western blot analysis; **c** gray value analysis of NKD1, β-catenin, c-myc, cyclinD1, and GAPDH protein bands; **d** the protein levels of NKD1, β-catenin, c-myc, and cyclinD1 as measured by western blot analysis; *p < 0.05 vs. the sh-NC or PBS groups; ^#^*p* < 0.05 vs. the sh-HOXA11-AS + sh-NC or sh-NC groups; ^&^p < 0.05 vs. the sh-HOXA11-AS + sh-NC group. All data are measurement data and are expressed as the means ± standard errors. Comparisons among multiple groups were performed with one-way analysis of variance. The experiments were repeated three times

It has been shown that HOXA11 can inhibit the Wnt signaling pathway^26^. To determine the potential relationship among HOXA11-AS, HOXA11, and the Wnt signaling pathway, HCC stem cells were treated with sh-HOXA11-AS, sh-HOXA11, DDK1, or PBS. The expression of HOXA11 and Wnt signaling pathway-related factors [naked1 (NKD1), β-catenin, c-myc, and cyclinD1] in each group was measured by western blot analysis. HOXA11 expression was significantly higher in the sh-HOXA11-AS + sh-NC group than in the sh-NC group, but was obviously lower in the sh-HOXA11-AS + sh-HOXA11 group than in the sh-HOXA11-AS + sh-NC group (Fig. 6a, b). In the DDK1 group, the expression of NKD1 was significantly upregulated, but the expression of β-catenin, c-myc, and cyclinD1 was obviously downregulated compared with that in the PBS group. This finding was consistent with the pattern in the sh-HOXA11-AS + sh-NC group compared to the sh-NC group. However, the opposite trend was found in the sh-HOXA11-AS+ sh-HOXA11 group compared to the sh-HOXA11-AS + sh-NC group (Fig. 6c, d). These results demonstrated that HOXA11-AS silencing can inactivate the Wnt signaling pathway by promoting HOXA11 expression in HCC stem cells.

### HOXA11-AS promotes HCC stem cell characteristics by activating the Wnt signaling pathway through HOXA11 downregulation

**Fig. 7:**
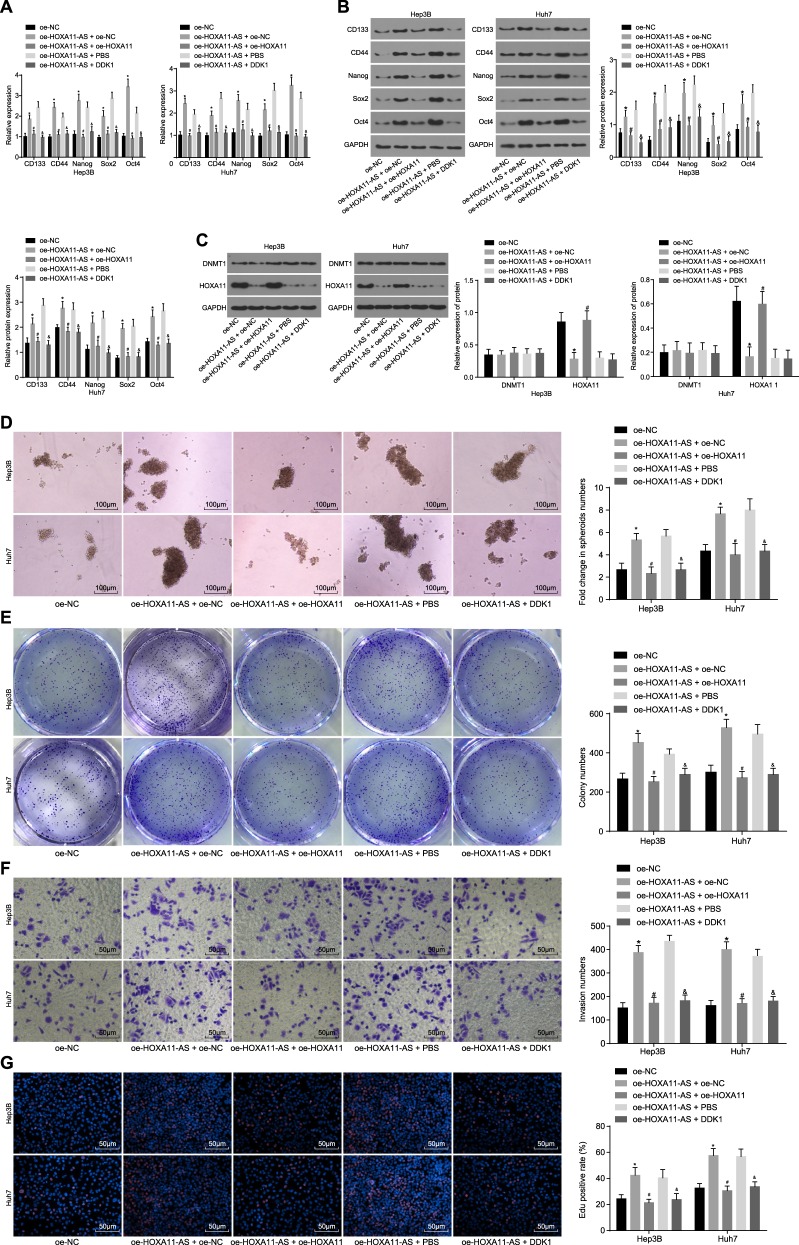
HOXA11 overexpression or Wnt signaling pathway inhibition reverses the promotive effect of HOXA11-AS on HCC stem cell characteristics. **a** The mRNA expression of CD133, CD44, Nanog, Sox2, and Oct4 in HCC stem cells as measured by RT-qPCR; **b** gray value analysis and protein levels of CD133, CD44, Nanog, Sox2, Oct4, and GAPDH in HCC stem cells; **c** gray value analysis and protein levels of HOXA11 and DNMT1 in HCC stem cells; **d** the tumorsphere formation ability of HCC stem cells following different treatments as evaluated by a tumorsphere formation assay (scale bar = 100 μm); **e** the colony formation ability of HCC stem cells as evaluated by a soft agar colony formation assay; **f** the invasion ability of HCC stem cells following different treatments as assessed by a Transwell assay (scale bar = 50 μm); **g** the proliferation ability of HCC stem cells as assessed by EdU staining (scale bar = 50 μm); *p < 0.05 vs. the oe-NC group; ^#^*p* < 0.05 vs. the oe-HOXA11-AS + oe-NC group; ^&^*p* < 0.05 vs. the oe-HOXA11-AS + PBS group. All data are measurement data and are expressed as the means ± standard errors; comparisons among multiple groups were performed with one-way analysis of variance. The experiments were repeated three times

The aforementioned results indicated that HOXA11-AS can regulate the Wnt signaling pathway by modulating HOXA11 in HCC stem cells; therefore, the effects of HOXA11-AS on the self-renewal, invasion, and proliferation abilities of HCC stem cells mediated via the HOXA11/Wnt signaling pathway axis were studied. HCC stem cells were successfully isolated from Huh7 and Hep3B cells and assigned to the oe-NC, oe-HOXA11-AS + oe-NC, oe-HOXA11-AS + oe-HOXA11, oe-HOXA11-AS + PBS, and oe-HOXA11-AS + DDK1 groups, and the expression levels of HOXA11, CDl33, CD44, Nanog, Sox2, and Oct4 in HCC stem cells after transfection were evaluated by RT-qPCR and western blot analyses. The expression levels of CDl33, CD44, Nanog, Sox2, and Oct4 were significantly higher, but that of HOXA11 was obviously lower in the oe-HOXA11-AS + oe-NC group than in the oe-NC group. However, the opposite results were found in the oe-HOXA11-AS + oe-HOXA11 group compared to the oe-HOXA11-AS + oe-NC group, as well as in the oe-HOXA11-AS + DDK1 group compared to the oe-HOXA11-AS + PBS group (*p* < 0.05), although HOXA11 expression showed no pronounced difference (Fig. 7a–c). Additionally, DNMT1 expression did not significantly differ among the groups. The self-renewal, invasion, and proliferation abilities of HCC stem cells were evaluated by tumorsphere formation assays, soft agar colony formation assays, Transwell assays, and EdU staining. The results showed that the number of tumorspheres, cell colonies, invaded cells, and proliferative cells were higher in the oe-HOXA11-AS + oe-NC group than in the oe-NC group, but were lower in the oe-HOXA11-AS + oe-HOXA11 group than in the oe-HOXA11-AS + oe-NC group, as well as in the oe-HOXA11-AS + DDK1 group compared to the oe-HOXA11-AS + PBS group (*p* < 0.05) (Fig. 7d–g). Together, these findings demonstrated that HOXA11-AS can activate the Wnt signaling pathway by inhibiting HOXA11 expression, thereby promoting HCC stem cell characteristics. Moreover, HOXA11 overexpression and Wnt signaling pathway inhibition were shown to reverse the promotive effect of HOXA11-AS on HCC stem cell self-renewal, invasion, and proliferation abilities.

### HOXA11 overexpression or Wnt signaling pathway inhibition reverses the promotive effect of HOXA11-AS on HCC stem cell characteristics

**Fig. 8:**
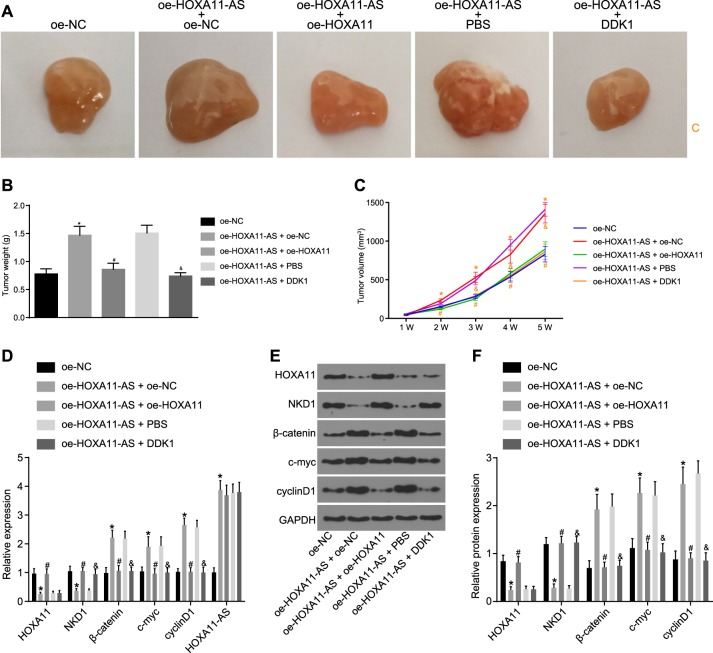
HOXA11 overexpression or Wnt signaling pathway inhibition reverses the promotive effect of HOXA11-AS on the tumorigenicity of HCC stem cells in vivo. **a** The growth nude mice subcutaneously injected with HCC stem cells with different treatments; **b** the weights of tumors obtained from nude mice injected with HCC stem cells with different treatments; **c** the volumes of tumors obtained from nude mice injected with HCC stem cells with different treatments; **d** the mRNA expression of HOXA11, NKD1, β-catenin, c-myc, and cyclinD1 in tumors from nude mice injected with HCC stem cells with different treatments as detected by RT-qPCR; **e**, **f** the protein expression of HOXA11, NKD1, β-catenin, c-myc, and cyclinD1 in tumors from nude mice injected with HCC stem cells with different treatments as examined by western blot analysis. *p < 0.05 vs. the oe-NC group; ^#^*p* < 0.05 vs. the oe-HOXA11-AS + oe-NC group; ^&^*p* < 0.05 vs. the oe-HOXA11-AS + PBS group. All data are measurement data and are expressed as the means ± standard deviations. Comparisons among multiple groups were performed with one-way analysis of variance or repeated measures analysis of variance (*n* = 6)

A xenograft tumor growth assay in nude mice was conducted to further validate the effect of HOXA11-AS on HCC stem cell characteristics mediated through the HOXA11/Wnt signaling pathway axis in vivo. An in vivo limiting dilution assay (LDA) assay was performed to measure the tumorigenicity of HCC stem cells in nude mice. The tumor formation rate was significantly higher in the oe-HOXA11-AS + oe-NC group than in the oe-NC group. The tumor formation rate was notably lower in the oe-HOXA11-AS + oe-HOXA11 group than in the oe-HOXA11-AS + oe-NC group and was reduced in the oe-HOXA11-AS + DDK1 group compared to the oe-HOXA11-AS + PBS group (all *p* < 0.05) (Table 4). The results also demonstrated that the tumor formation time was shorter, the tumor growth rate was faster, and the tumor volume was larger in the oe-HOXA11-AS + oe-NC group than in the oe-NC group. Conversely, the tumor formation time was longer, the tumor growth rate was slower, and the tumor volume was smaller in the oe-HOXA11-AS + oe-HOXA11 group than in the oe-HOXA11-AS + oe-NC group. A similar tendency was also found in the oe-HOXA11-AS + DDK1 group compared to the oe-HOXA11-AS + PBS group (all *p* < 0.05) (Fig. 8a–c). These results demonstrated that HOXA11 overexpression or Wnt signaling pathway inhibition can reverse the promotive effect of HOXA11-AS on HCC stem cell characteristics in vivo. Subsequently, RT-qPCR and western blot analyses were employed to measure the expression levels of HOXA11 and Wnt signaling pathway-related factors (NKD1, β-catenin, c-myc, and cyclinD1). The DDK1 group exhibited significantly higher expression of NKD1, but obviously lower expression of β-catenin, c-myc, and cyclinD1 than the PBS group. In addition, a pronounced increase in the expression of HOXA11-AS, β-catenin, c-myc, and cyclinD1, but a significant reduction in the expression of HOXA11 and NKD1 was found in the oe-HOXA11-AS + oe-NC group relative to the oe-NC group. Compared with the oe-HOXA11-AS + oe-NC group, the oe-HOXA11-AS + oe-HOXA11 group showed a considerable decline in the expression of β-catenin, c-myc, and cyclinD1, but a marked increase in the expression of HOXA11 and NKD1. Compared with the oe-HOXA11-AS + PBS group, the oe-HOXA11-AS + DDK1 group exhibited significantly decreased expression of β-catenin, c-myc, and cyclinD1, but increased expression of NKD1 and no change in HOXA11 expression (Fig. 8d–f).

**Table 4:** Tumorigenicity of Hep3B and Huh7 spheres

Injected cells	oe-NC	oe-HOXA11-AS + oe-NC	oe-HOXA11-AS + oe-HOXA11	oe-HOXA11-AS + PBS	oe-HOXA11-AS + DDK1
5 × 10^3^	3/6	6/6	1/6	6/6	2/6
1 × 10^4^	2/6	5/6	3/6	6/6	1/6
5 × 10^4^	5/6	6/6	2/6	4/6	4/6
1 × 10^5^	4/6	6/6	4/6	6/6	5/6
Total	14/24 (58.33%)	23/24 (95.83%)*	10/24 (41.67%)^#^	22/24 (91.67%)	12/24 (50.00%)^&^

Note: *p < 0.05 vs. the oe-NC group; ^#^*p* < 0.05 vs. the oe-HOXA11-AS + oe-NC group; ^&^*p* < 0.05 vs. the oe-HOXA11-AS + PBS group

*HOXA11-AS* homeobox A11 antisense, *HOXA11* homeobox A11, *NC* negative control, *PBS* phosphate-buffered saline, *DDK1* Dickkopf-1

## Discussion

HCC remains one of the top causes of cancer-related deaths worldwide, especially in sub-Saharan Africa and eastern Asia^5^. Extensive treatments such as resection, tumor ablation, chemotherapy, and liver transplantation are restricted to patients with advanced liver cancer, and recurrence is frequently noted^28^. Therefore, a comprehensive understanding of the molecular pathology underlying hepatic carcinogenesis is essential to advance disease prognosis prediction and therapeutics. In this study, we demonstrated the oncogenic role of HOXA11-AS in HCC. HOXA11-AS negatively regulated HOXA11 by epigenetic modulation, whereas HOXA11-AS silencing inactivated Wnt signaling and inhibited the self-renewal, invasion, and proliferation of HCC stem cells.

A key finding of the present study was that the lncRNA HOXA11-AS was highly expressed in HCC cell lines and HCC stem cells, as well as in tumor tissues from HCC patients; concurrently, the expression of HOXA11 was decreased. HOXA11-AS not only represses HOXA11 but also regulates other coding genes in HCC. For example, HOXA11-AS was shown to inhibit DUSP5 transcription and promote HCC cell proliferation by regulating the cell cycle and apoptosis^29^. Other studies have noted that the inhibition of LATS1 by HOXA11-AS enhanced the proliferation of HCC cells via binding of the enhancer protein EZH2^30^. Considering the current findings, it appears to be confirmed that HOXA11-AS is a promoter of the HCC cell cycle. In agreement with this assertion, previous studies have suggested a similar function of HOXA11-AS in multiple cancers. In glioma, overexpression of HOXA11-AS promotes cell proliferation, while HOXA11-AS silencing inhibits cell cycle progression^31^. HOXA11-AS was found to be highly expressed in non-small-cell lung cancer and was shown to promote cell proliferation and invasion via sponging miR-124^32^. Similarly, HOXA11-AS functions as a sponge of miR-140-5p and promotes glioma tumorigenesis via regulation of the cell cycle and apoptosis^33^. Considering these observations, we conclude that HOXA11-AS has an oncogenic role in most types of cancers and that HOXA11-AS can regulate the cell cycle via downregulation of coding genes or miRNAs.

It was found that overexpression of HOXA11-AS increased the levels of the membrane markers CD133 and CD44, as well as the transcription factors Nanog, Sox2, and Oct4. All of these proteins are recognized as stem cell markers. In cervical cancer, HOXA11-AS silencing was shown to decrease the expression of matrix metalloproteinase-2 (MMP-2), MMP-9, and vascular endothelial growth factor and increase the number of CD133^+^CD44^+^ cells, indicating an increase in the cancer stem cell population^14^. In adipose tissues, suppression of HOXA11-AS inhibited adipogenesis-related genes and blocked adipocyte differentiation, causing decreased lipid accumulation in human adipose-derived stem cells^34^. In summary, considering the previous evidence, we conclude that HOXA11-AS promotes the differentiation of stem cells. HOXA11 is critical to vertebrate embryonic development^35^, and it is speculated that HOXA11-AS might regulate HOXA11 expression during this stage. Both embryonic development and stem cell growth involve rapid cell proliferation. The Wnt signaling pathway participates in axis patterning and in cell differentiation, proliferation, and migration^36^. The downstream targets regulated by HOXA11-AS appear to affect the Wnt signaling pathway, further indicating that lncRNA function is integral to both embryonic development and tumorigenesis.

HOXA11-AS regulates its target genes by epigenetic methylation. It was confirmed that HOXA11-AS recruited the DNMT1 enzyme to the promoter region of the *HOXA11* gene and increased HOXA11 methylation in HCC. Previous studies have shown similar epigenetic regulation of HOXA11-AS in other cancers. In non-small-cell lung cancer, HOXA11-AS guides EZH2 and DNMT1 proteins to the promoter region of miR-200b and blocks miR-200b transcription^37^. Similarly, HOXA11-AS is able to recruit EZH2 via the DNMT1 methyltransferase complex to regulate miR-1297 and promote gastric cancer cell proliferation and invasion^27^. Regarding methylation, HOXA11-AS was previously shown to repress the *DUSP5* gene by recruiting EZH2 to the promoter region of DUSP5^29^. EZH2 is a polycomb group protein, and the PRC2/EED-EZH2 complex methylates H3K9 and H3K27, causing transcriptional repression of target genes^38^. EZH2 also modulates DNA methylation via binding DNMT1 and recruiting it to DNA^39^. DNMT1 and EZH2 methylate distinct residues, but result in similar target gene repression.

Analysis of microarray data from HOXA11 reexpression experiments identified various DEGs related to the Wnt signaling pathway, such as wnt6 and NKD1. NKD1 expression was positively correlated with HOXA11; in addition, reexpression of HOXA11 induced significant increases in NKD1 mRNA and protein expression, and vice versa^26^. NKD is a negative regulator of Wnt signaling; its upregulation inhibits canonical Wnt/β-catenin signaling during early development in zebrafish and exacerbates the cyclopia and axial mesendoderm convergence and extension defect in the noncanonical Wnt/PCP mutant silberblick (slb/wnt11)^40^. NKD1 was also noted to interact with Dsh, an intracellular regulator of canonical Wnt signaling and planar cell polarity (PCP) pathways, consequently suppressing Wnt signaling and stimulating the c-Jun N-terminal kinase, a component of the PCP pathway^41^.

**Fig. 9:**
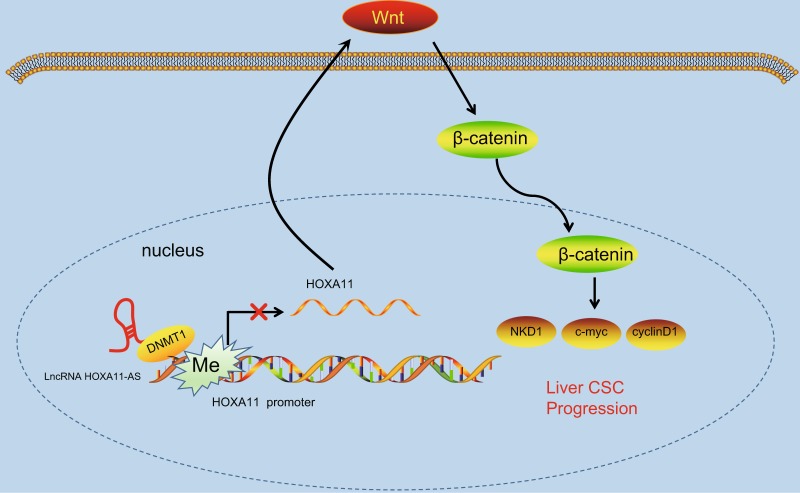
The mechanism by which lncRNA HOXA11-AS regulates stem cell characteristics in hepatocellular carcinoma via the modulation of HOXA11 gene expression and the Wnt signaling pathway. The lncRNA HOXA11-AS recruits DNMT1 to the promoter region of HOXA11, causing its methylation and subsequently resulting in suppression of HOXA11 expression and activation of the Wnt signaling pathway, which ultimately promotes stem cell characteristics in hepatocellular carcinoma

In conclusion, the results from our study provide evidence that HOXA11-AS silencing promotes HOXA11 expression and inhibits the Wnt signaling pathway, subsequently reducing HCC stem cell proliferation, invasion, and self-renewal (Fig. 9). These findings demonstrate a novel regulatory mechanism by which HOXA11-AS recruits DNMT1 to methylate the HOXA11 promoter, thus repressing HOXA11 transcription. The HOXA11-AS regulatory axis may contain promising molecular targets for the future treatment of HCC. Further studies are warranted to validate this mechanism of HOXA11-AS regulation in other cancers.

## Data Availability

The datasets generated/analyzed during the current study are available.
